# Norms and Social Network–Centric Behavior Change Intervention (Nam Nalavazhvu) for Improved Toilet Usage in Peri-Urban Communities of Tamil Nadu: Protocol for a Cluster-Randomized Controlled Trial

**DOI:** 10.2196/24407

**Published:** 2021-05-03

**Authors:** Sania Ashraf, Cristina Bicchieri, Maryann G Delea, Upasak Das, Kavita Chauhan, Jinyi Kuang, Alex Shpenev, Erik Thulin

**Affiliations:** 1 Center for Social Norms and Behavioral Dynamics University of Pennsylvania Philadelphia, PA United States; 2 Gangarosa Department of Environmental Health Rollins School of Public Health Emory University Atlanta, GA United States; 3 Hubert Department of Global Health Rollins School of Public Health Emory University Atlanta, GA United States; 4 Global Development Institute University of Manchester Manchester United Kingdom; 5 Center for Behavior and the Environment, Rare Arlington, VA United States

**Keywords:** sanitation, behavior change, social norms, toilet

## Abstract

**Background:**

Inconsistent toilet usage is a continuing challenge in India. Despite the impact of social expectations on toilet usage, few programs and studies have developed theoretically grounded norm-centric behavior change interventions to increase toilet use in low-income settings.

**Objective:**

The objective of this paper is to detail the rationale and design of an ex ante, parallel cluster-randomized trial evaluating the impact of a demand-side, norm-centric behavior change intervention on exclusive toilet use and maintenance in peri-urban Tamil Nadu, India.

**Methods:**

Following formative research, we developed an evidence-based norm-centric behavior change intervention called Nam Nalavazhvu (Tamil for “our well-being”). The multilevel intervention aims to improve toilet usage by shifting empirical expectations or beliefs about other relevant people’s sanitation practices. It also provides action-oriented information to aid individuals to set goals and overcome barriers to own, consistently use, and maintain their toilets. This trial includes 76 wards in the Pudukkottai and Karur districts, where half were randomly assigned to receive the intervention and the remaining served as counterfactuals.

**Results:**

We enrolled wards and conducted a baseline survey among randomly selected individuals in all 76 wards. The 1-year behavior change intervention is currently ongoing. At the endline, we will collect relevant data and compare results between study arms to determine the impacts of the Nam Nalavazhvu intervention on sanitation-related behavioral, health, and well-being outcomes and potential moderators. This study is powered to detect differences in the prevalence of exclusive toilet use between study arms. We are also conducting a process evaluation to understand the extent to which the intervention was implemented as designed, given the special pandemic context.

**Conclusions:**

Findings from this trial will inform norm-centric behavior change strategies to improve exclusive toilet usage.

**Trial Registration:**

ClinicalTrials.gov NCT04269824; https://www.clinicaltrials.gov/ct2/show/NCT04269824

**International Registered Report Identifier (IRRID):**

DERR1-10.2196/24407

## Introduction

### Study Rationale

Open defecation practices enable environmental contamination and contribute to poor health, well-being, and safety globally [1-3]. In the past decade, national sanitation programs, such as the Swachh Bharat Mission (SBM), have significantly increased coverage of private, shared, and public toilets to end persistent open defecation practices in India [4,5]. In addition, they have promoted exclusive use or using a toilet every time for defecation. Despite increased access to toilets, in many communities, complex sociocultural norms, along with technological and financial barriers, prevent individuals from using a toilet every time or exclusively using a toilet for defecation purposes [6-8]. Recent behavior change interventions designed to promote toilet use in rural India yielded, on average, a 5% increase in reported use amongst toilet owners, which is comparable to results generated by SBM [9-11]. Sustaining exclusive toilet use among all household members is a national priority for the current SBM 2.0, also known as the Open Defecation–Free plus scheme [12]. This is also aligned with the United Nations’ Sustainable Development Goal 6.2, which calls on countries to “achieve access to adequate and equitable sanitation and hygiene for all and end open defecation” by 2030 [13].

Numerous studies have highlighted the importance of social beliefs and preferences on toilet construction and adoption [14-17]. Studies based in India found that several factors such as sociocultural inequalities, access to resources, and psychosocial determinants, such as perceived use among others in one’s community, impacted toilet ownership and use [11,18-20]. Social networks were also found to be relevant: a study in rural Karnataka showed that individuals were more likely to own toilets if their social contacts owned one [21]. Following the achievements of SBM, in a context of high toilet ownership and use, perceptions of others’ toilet ownership and approval might be particularly important motivators of toilet-related behavior change in India [11,19].

Previous studies that assessed psychosocial determinants of toilet use and included norm-based messaging to promote toilet use in India were primarily guided by Community-Led Total Sanitation (CLTS); the Risks, Attitudes, Norms, Abilities, and Self-regulation (RANAS) approach; or Behavior Centered Design (BCD). These approaches include norms as one driver of behavior but do not systematically evaluate and leverage specific social expectations that influence the norm [11,22-24]. It is not well understood whether the collective behavior of toilet usage is conditional on social expectations held by individuals. Social norms theory (SNT) uses a novel norms diagnostic approach to understand social expectations in distinguishing between independent or socially interdependent behaviors [25]. We based our theoretical investigation on SNT to conduct formative research as the first part of the Longitudinal Evaluation of Norms and Network Study (LENNS). We specifically investigated whether toilet use behavior is driven by beliefs that most other people are using one (empirical expectations) or whether others think one should use it (normative expectations) [26]. By measuring social expectations and other social determinants, we determined that toilet use is socially conditional on empirical expectations in these communities (further details in following section). This allowed us to leverage specific normative components to focus our behavior change strategy.

This study protocol summarizes the rationale and methods of a cluster-randomized trial (CRT; LENNS) that aims to evaluate the impact of a multilevel, demand-side behavior change intervention package called Nam Nalavazhvu on exclusive toilet use and maintenance. Prior studies have not explored norm-based intervention techniques specifically designed to change empirical expectations of sanitation behaviors in low-income communities. Findings from this multilevel intervention can be used to adapt them for other communities to shift norms around toilet use. There is limited evidence on whether intervening upon empirical expectations of others’ sanitation behaviors can lead to the emergence of normative expectations of toilet use. This type of phenomenon is theoretically plausible, and if demonstrated through this study, our findings can lead to insights to inform the design of norm-focused behavior change strategies [27,28]. Finally, our study is based in peri-urban communities, which will add to the currently limited sanitation literature regarding interventions that benefit peri-urban populations [29-31].

### Specific Aims

The primary research aim of this study is to evaluate the impact of the Nam Nalavazhvu intervention on behavioral and health outcomes. The aim specifically focuses on the following: exclusive toilet usage, defined as reported use of a toilet every time for defecation among individuals aged 5 years and older (primary outcome).

Secondary aims include assessing the impact of the intervention on the following: access to improved toilets (individual or shared) for households without a toilet; maintenance of sanitation facilities for sustained use; empirical expectations, normative expectations, and other behavioral antecedents; mental well-being of respondents; and diarrheal outcomes in all household members and respiratory health in children under 5 years.

## Methods

### Study Setting

**Figure 1:**
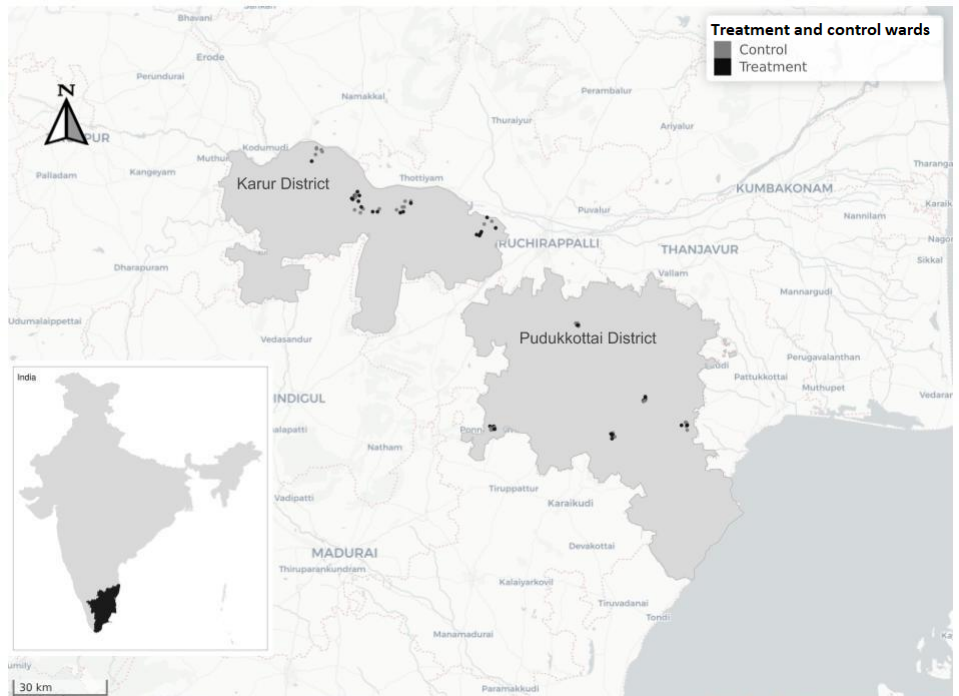
Study sites in Karur and Pudukkottai districts of Tamil Nadu, India.

The study is being conducted in 76 wards in peri-urban areas of Pudukkottai and Karur districts in Tamil Nadu, India (Figure 1). The unit of randomization for this study was the ward, the smallest administrative unit of a town panchayat. According to the 2011 Census, these are urbanizing districts, where in Pudukkottai and Karur respectively, the residents were mainly agricultural laborers (31% and 34%, respectively), workers in industries (1.3% and 1.2%, respectively), and other private businesses (16% and 43%, respectively). These districts consist primarily of Hindus (88% and 93%, respectively) and a minority of Muslims (7.1% and 5.1%, respectively). Although both districts were declared open defecation–free in October 2019, there were variations in toilet coverage and use across constituent wards, which we captured through informal conversations with officials.

### Study Design

The LENNS trial is an ex ante, parallel CRT. We will collect data relevant to our research questions in both study arms and compare results between the intervention and counterfactual arms to determine the impact of the intervention on the primary and intermediate outcomes. This study is powered to detect differences in the prevalence of exclusive toilet use between study arms.

Clusters are defined as wards within purposively selected town panchayats. Half of the clusters were randomized to receive the Nam Nalavazhvu intervention, while the other half will not receive any active intervention as part of the study. Prior to our enrollment of study participants, we created a buffer zone, minimally one ward in distance, between study clusters to minimize spillover. As this study is being conducted in a context where the Indian government is actively implementing SBM, both study arms may be subject to government-led sanitation program activities.

**Figure 2:**
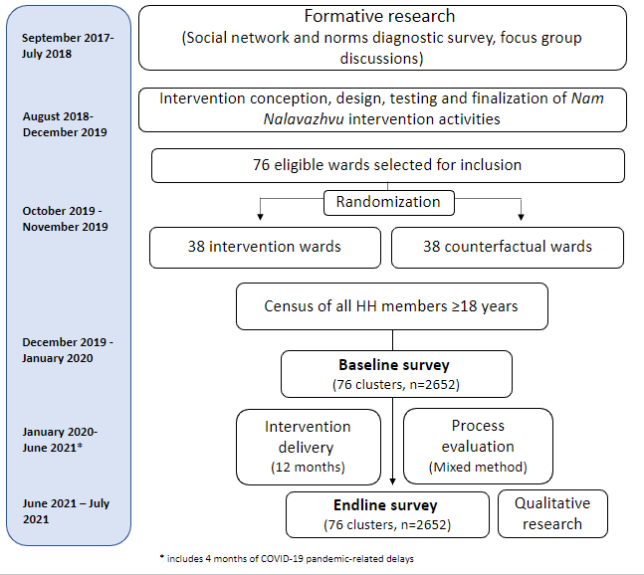
Summary of overall study design, including the timeline for impact evaluation to assess the effectiveness of the Nam Nalavazhvu intervention in Tamil Nadu, India. HH: household.

The Nam Nalavazhvu intervention is further detailed in the following section. We co-designed this intervention with our implementation partner, a local nongovernmental organization called Swasti. Prior to the CRT, we conducted a 3-month trial of improved practices to test, refine, and revise our behavior change intervention activities and materials among a separate population in the same study districts (Figure 2). In the CRT, the intervention will be implemented for 12 months. A baseline and 1-year follow up survey will be used to assess the impact of the intervention. We will also conduct a process evaluation to determine the extent to which the intervention was implemented as designed and identify successful pathways or barriers to the adoption of improved sanitation behaviors. Our process evaluation will assess fidelity of intervention implementation, reach, and contextual changes in community and household conditions that may facilitate improved behavioral adoption and outcomes. In addition, we will also conduct qualitative research with respondents and stakeholders to assess exposure and dose received of the intervention. Further, we aim to assess the extent of spillover in the control and the adjacent wards using mixed-method research tools.

### Rationale and Formative Research

The Nam Nalavazhvu intervention design leverages 2 years of mixed-method formative research which included 2 rounds of surveys with the following aims: (1) map social networks in similar communities and (2) systematically diagnose the collective behavior of toilet use with SNT. These sequential assessments were conducted concurrently in both Bihar and Tamil Nadu [26]. Specifically, the formative research comprised the following: a social network survey (n=3370) to understand the size, structure, and nature of social ties related to sanitation behaviors and ownership; 18 focus group discussions with men and women, including young unmarried women and older women to explore social and gender norms related to toilet construction and use; and a social norms survey (n=5052) to assess social beliefs, expectations, and determinants of open defecation.

As mentioned, the Nam Nalavazhvu intervention is based on the SNT, which highlights the role of social expectations and conditional preferences in guiding collective behaviors [25]. We drew on previous literature and investigated known social factors, such as preference for open defecation, perceived barriers related to toilet ownership and maintenance, and implications of social expectations of others in one’s community [32,33]. We measured social beliefs, along with empirical (what others in their community do) and normative expectations (beliefs about what others should do), related to toilet usage and used the results to assess if toilet use was a socially interdependent behavior in Indian communities. Using vignettes and regression analyses, we found that empirical expectations were a strong driver of toilet use, while normative expectations were not [26]. This suggested toilet use in this context was a “descriptive norm” or an interdependent behavior where beliefs of what most other people do influence one’s behavior [25]. This is consistent with other recent studies that reported empirical expectations as a significant psychosocial determinant for toilet ownership [11,15]. Based on these findings, we designed a theoretically grounded, evidence-based behavior change intervention. We will evaluate its effectiveness on the uptake of exclusive toilet use and maintenance through this randomized trial.

### Theory and Evidence-Based Intervention Design

Empirical expectations can be powerful drivers of human behavior [34,35]. Norm nudging is a technique that aims to change the social expectations of an improved behavior of those around the respondent, preferably relevant social members [27]. The key assumption is that compliance is conditional or dependent on this change in expectation. Previous research to improve proenvironmental behaviors showed that telling individuals about their neighbors’ electricity consumption reduced their own usage [36]. Using similar techniques, another study successfully nudged hotel guests to reuse towels during their stay [37]. Evidence also suggests that descriptive norm messages are more effective when provided through personalized normative feedback than through broadcasting [38-40].

**Figure 3:**
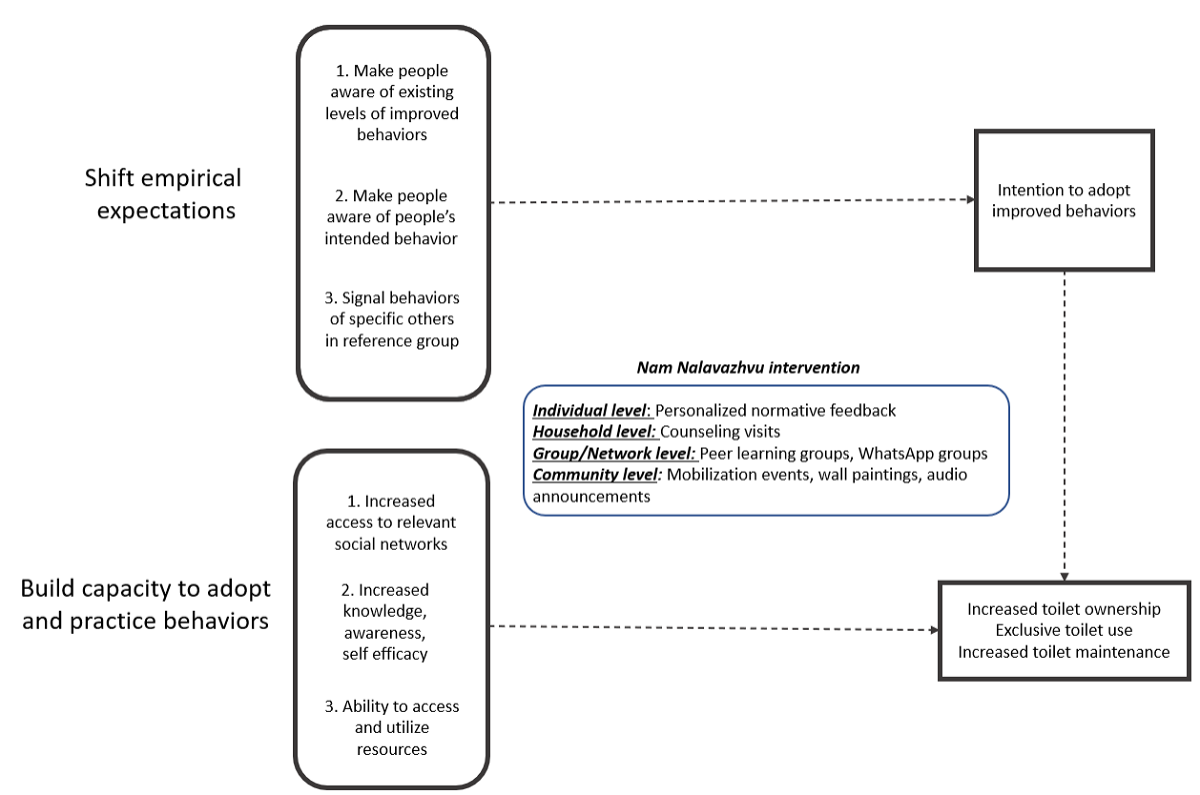
Theory of change for the Nam Nalavazhvu behavior change intervention, Tamil Nadu, India 2020

We synthesized the insights from our norm and social network assessment in Tamil Nadu to employ a systematic, multistep process to design the Nam Nalavazhvu intervention and used a theory of change approach to do so [41]. We used problem and solution tree analysis to depict causal streams of open defecation practices, including the sanitation-related social norms that supported these practices. We used theory and evidence to articulate the mechanisms through which change and maintenance of improved behaviors may occur. Based on the specific behavioral factors, we developed an intervention-mapping matrix and identified potential intervention techniques guided by Michie et al [42]. We acknowledged that norm compliance for individuals is influenced by household, community, and contextual factors by using a socioecological framework [43]. By 2019, SBM had increased coverage of toilets and had declared most states open defecation free [4]. We leveraged this context to disseminate descriptive norms information about those with improved sanitation practices in one’s community to shift empirical expectations and encourage others to conform to the norm of toilet use. We intentionally did not focus on techniques that use injunctive norms or perceived disapproval of others due to potential unintended negative consequences; to leverage personalized normative messages, we included techniques to engage community members across gender and age groups (Figure 3) [26].

### Description of Intervention

Nam Nalavazhvu means “our well-being” in Tamil and reflects a demand-side, norm-centric intervention designed to improve exclusive use of sanitation facilities for defecation purposes. Intervention activities focus on shifting empirical expectations by broadcasting improved sanitation behavior of relevant others in the community through activities at all levels and capacity building and increased action knowledge through expanded social networks and access at the group level. The program will not directly provide hardware or build toilets but actively address opportunity limitations. Details of the intervention design process as well as the theoretical and behavioral framework will be published in separate forthcoming articles.

This multilevel intervention includes activities at the individual, household, group, and ward levels (Textbox 1).

**Nam Nalavazhvu intervention activities for the peri-urban area, Tamil Nadu.**

Ward level

- Community mobilization and public commitment events
- Mass audio broadcasting descriptive information
- Wall paintings promoting toilet ownership and use

Group level

- Peer learning sessions
- Descriptive norm messages delivered to social network members via community influencers

Household level

- Tailored counseling sessions with household members
- Visual signals of improved sanitation practices

The individual level, in concurrence with household counseling sessions

- Personalized advice, including information regarding similar others’ practices

### Descriptive Information Dissemination and Capacity Building

To update empirical expectations regarding others’ improved sanitation practices, we will periodically collect information about sanitation practices from all households in study clusters. We will then disseminate related descriptive norm information during community events, household visits, and via text messages from peers in social groups. Action knowledge and social connections between ward members will be increased by connecting neighbors, outreach workers, and similar individuals in the ward who have adopted improved sanitation practices through events and activities. Information about available financial schemes, sanitation markets, and masons will be provided, and the outreach workers will assist in setting goals and taking steps to make progress towards them. Details of the activities are described in the following sections.

### Ward Level

Prior to the intervention delivery, ward outreach workers will meet local stakeholders to conduct a social and resource mapping to engage them, foster support for the project goals, and identify influencers who can be leveraged during the activities. Preliminary meetings will be conducted with these influencers (eg, teachers, religious leaders, self-help group coordinators) to mobilize them. Ward outreach workers will also leave their contact cards to establish communication channels.

#### Roving Audio Announcements

Automobiles with loudspeakers will announce the launch of the program and invite community members to group sessions and community events. The audio content will include customized jingles to promote toilet use and later disseminate ward level data regarding people’s actual and intended sanitation practices.

#### Wall Paintings

Wall paintings (6 × 4 feet) will be used in at least four public locations per ward. Permissions will be secured from required private or government officials to paint the publicly visible wall. The motto, tagline, and norm-centric images will be painted to encourage others to “join the proud toilet owners.”

#### Community Mobilization and Commitment Events

Participants will include influential political and community leaders to applaud exclusive toilet users and promote improved sanitation practices. Messages will highlight the change happening in their communities and the benefits of using a toilet for families, and facilitate a public commitment to exclusive toilet use. These messages will reinforce those heard by residents through other group and household activities.

### Group Level

#### Peer Learning Session

Six to eight sex-segregated groups will be convened in each ward over 1 year to facilitate social networking between toilet users and nontoilet users. The outreach worker’s supervisor will facilitate these 60-90–minute sessions. The facilitator will use social and behavior change communication materials (eg, story cards, video content) to enable norm-focused conversations and information sharing among group members on the access of sanitation markets, barrier identification, planning, and coping by discussing personal experiences, challenges, and solutions regarding target sanitation practices. Peer-to-peer knowledge sharing will be encouraged.

#### Social Media to Reference Networks

Influential community members who are active on social media will be recruited as volunteer promoters or advocates. We will invite them to use their social media groups to send key messages promoted by the project. The LENNS team will develop messages and monitor their deployment as per the schedule. These advocates will also use their social groups to broadcast testimonials from toilet adopters, and related community events and activities.

### Household Level

#### Household Counseling Visits

Household counseling will allow outreach workers to acquire information about the household’s potential for change. The outreach workers will communicate ward-specific toilet use of similar households to motivate change. Flipbooks and story cards will be used to engage and motivate household members. The outreach worker will counsel them on how to achieve their goals by mapping out the next steps using goal cards. These households will also be informed of their neighbors’ improved practices. Ward outreach workers will focus on those lagging behind to motivate them to change, pointing out others like them who already have.

#### Visual Signals of Improved Practices

Improved behaviors of neighbors will be signaled using bright decals or stickers, placed in public view on the household wall. These aim to serve as a goal and a source of pride for the households who receive and display them.

During the trial of improved practices, we incorporated feedback from community members and pilot intervention recipients to ensure the feasibility, appropriateness, and acceptability of the content, and the delivery mechanism of the Nam Nalavazhvu intervention activities. Notably, we confirmed that signaling toilet ownership and use through stickers, household visits, group meetings, and testimonials were acceptable and perceived as encouraging and aspirational. We ensured that all content used positive framing and included engagements with community stakeholders to collaboratively host group and community events. We also assessed whether incentives were necessary for the influencers, and found they preferred nonmonetary compensation or incentives. We plan to recognize them at community events for their work, present them with a volunteer experience certificate, and invite them to group sessions held as a part of the intervention activities.

The implementation team is hired and managed by Swasti. Investigators at the Center for Social Norms and Behavioral Dynamics (CSNBD) at the University of Pennsylvania will provide training, technical input, and oversight to the implementation process. The main delivery agent will be ward outreach workers who are residents of the ward with a minimum of 12 years of formal education. Field supervisors will help organize and facilitate group and ward-level activities. In addition, the study will include influential members from the community to disseminate promotional and motivational messages to their social networks.

Participation in these activities will be voluntary. The audio announcements and visual cues or paintings in public spaces will be apparent during the intervention. Ward outreach workers will consider any requests to adjust the recipient’s level engagement in the activities to ensure the consent and comfort of participants.

### Public Involvement

As mentioned in the intervention details, members of the public will be used to disseminate promotional messages through social media, using decals, active participation in groups and community events. During the pilot phase, we also incorporated their feedback for improving intervention delivery techniques and platforms prior to the trial.

### Eligibility Criteria

We randomly selected 5 town panchayats from each district. Researchers visited several wards with local Swasti officials to ensure they reflected the generalized peri-urban setting. To identify potential study wards within these town panchayats, we met local executive officers to assess ward maps. We used the following exclusion criteria: commercialized wards with few residential households, urbanized wards with known high or complete coverage of improved toilet access according to town panchayat official records, and wards which bordered two or more adjoining wards (to reduce spillover).

Our sampling frame excluded town panchayats where we piloted our interventions (n=3) or those where we did not receive permission to proceed due to political concerns (n=1).

The unit of randomization for this CRT was the ward. The demarcation of the ward was derived from the 2011 census conducted by the Ministry of Home Affairs of the Government of India [34]. Trained field teams generated ward maps to delineate the ward boundaries, confirmed it with residents, and ensured a buffer zone. We also engaged ward-level stakeholders to assist in the mapping process and gain access to communities. From the ward maps, we identified the adjoining wards, which share the borders and excluded 1 from the list of potential wards to ensure we reduced the possibility of spillover between clusters. Of the total of 153 wards considered in 10 town panchayats, we found 79 eligible wards based on our criteria. We selected a random sample of 76 wards to include in our study. These wards are more representative of residential peri-urban wards in Tamil Nadu. As the treatment arm was randomly selected, we do not expect to find systematic differences between the treatment and control arms. Following this exercise, a minimum of 1 km distance was ensured between the residential units in each cluster.

All ward residents from intervention clusters are eligible to participate in the intervention activities. For data collection, field workers surveyed randomly selected household members who were 18 years or above, planned to reside in that household continuously for the next year, and were willing and able to participate.

### Selection and Assignment of Interventions

Following the baseline data collection, a coinvestigator (UD) randomly assigned the wards to counterfactual and intervention arms in a 1:1 ratio, using a computer-generated randomization sequence. The randomization was geographically pair-matched within each town panchayat (Multimedia Appendix 1). Balancing the study arms across geography will allow us to adjust for spatially clustered features or events that may be associated with our outcome. Given the nature of the intervention, the study investigators and the participants will not be masked to the intervention assignment. The data collection team will be masked to the treatment assignment.

### Recruitment

Trained enumerators conducted a listing exercise to generate a sampling frame of eligible individuals within each ward. Enumerators approached the household and asked the selected individuals if they would consent to participate in the study. If the selected individual was absent or unavailable, up to three repeat visits were made to enroll them. If unsuccessful, the enumerators approached the next randomly selected individual from that cluster. Following randomization, households in intervention wards were enrolled following an oral consent process.

The individuals enrolled in qualitative studies will be purposively selected from intervention wards to address relevant research questions. Those sampled from the counterfactual wards will allow us to investigate spillover of the Nam Nalavazhvu intervention.

### Sample Size

This study is powered to detect differences in the prevalence of exclusive toilet use between study arms. Measuring exclusive use is problematic due to recall and desirability bias. As a result, during our formative research phase, we asked individuals about their defecation place the last time they needed to defecate during 2 rounds of data collection conducted 8 months apart. We incorporated the correlation between these 2 measures in our sample size calculation. We powered our study at 80% based on the prevalence of reported toilet use during last defecation in peri-urban Tamil Nadu (estimated at 64.4% in 2018) and assumed a 10-percentage-point improvement as the minimum important effect [26]. To detect such a minimal effect in exclusive toilet use (given an observed intracluster correlation of 11% and correlation of last use with the one measured in the fall of 2017 of 47.5%) [26], we estimated a requirement of 76 clusters (38 clusters per arm). We require 30 individuals per cluster. Assuming 10% loss to follow-up, we will engage 34 individuals per cluster for a total of 2280 individuals. As we will collect household level toilet usage data, we will have data for more individuals beyond the actual number of respondents.

### Study Outcomes and Measures

We will evaluate these outcomes at baseline and at endline in both the intervention and counterfactual clusters 1 year after the intervention implementation using verbal surveys. We will characterize sanitation facilities using standardized categorizations through direct observations.

#### Primary Outcome

The primary outcome will be the proportion of households where all members (18 years or older) exclusively use a toilet every time they defecate. We will combine responses to several self-reported toilet use behaviors to determine exclusive toilet use in the previous 2 days. We will also observe toilets to check for signs of use (Multimedia Appendix 2).

#### Secondary Outcomes

There will be 5 secondary outcomes: (1) presence and access to improved toilets will be assessed using standard questions, while spot observations of toilets will be used to assess maintenance, functionality, and recent use; (2) mental well-being will be measured using The 5-item World Health Organization Well-Being Index (WHO-5); (3) diarrheal disease for all household members will be measured using the WHO definition of three or more loose stools in a 24-hour period, with or without the presence of blood; (4) respiratory illness for children under 5 years will be measured using reported cough and/or difficulty breathing or shortness of breath according to the WHO’s Integrated Management of Childhood illness; and (5) intermediate behavioral antecedents such as empirical expectations (ie, what other people do), normative expectations (ie, what other people think one should do) of prevalence of toilet ownership, exclusive use, and maintenance will be measured using tested indicators (see Multimedia Appendix 2)

### Data Collection

Randomly selected individuals were enrolled for the impact evaluation. They responded to a baseline survey and will be approached to complete a 1-year follow-up survey administered by trained enumerators. The enumerators will be masked to the intervention assignment. However, given the nature of the intervention, they might observe Nam Nalavazhvu intervention products in the household during the follow-up survey.

We enrolled 34 respondents from each cluster and will attempt to reinterview them at endline. We will consider respondents lost to follow-up if any of the following occur: they refuse to participate in the follow-up survey, they relocate elsewhere outside the intervention ward, or the field team is unable to reach them after 3 attempts during data collection. We will replace respondents lost to follow-up by recruiting additional respondents from the sampling frame of the ward. We will ensure 34 respondents per ward at the endline to have adequate power to conduct cross-sectional posttest design analysis.

CSNBD researchers will work with trainers to conduct a 10-day training session prior to each survey round. The training sessions will be conducted in Tamil. Prior to the baseline survey, the instrument was administered to respondents similar to the target group to ensure comprehension. We incorporated feedback to clarify language, framing, answer choices, and administration of the survey. The survey data were collected using personal handheld devices. These electronic surveys were tested to address issues with data capture, skip patterns, and validity checks for each item in a pilot study. Quality assurance steps were taken to improve data accuracy and included regular field-level data checks and dual data capture of objective measures from a subset of households by field supervisors. Researchers from CSNBD also visited the study sites randomly to assess the situation pertaining to the survey. Weekly phone meetings were conducted with the data collection agency to ensure the quality of data.

The implementation partner will collect routine monitoring data to capture information about intervention fidelity and exposure. We will take steps to ensure that they do not involve participants enrolled in the impact evaluation to reduce participant fatigue.

We will conduct 3 rounds of qualitative data collection as part of the process documentation for this study. These will be at 3 months, 6 months, and 1 year after the start of the intervention and will assess participant response, acceptability, and the beliefs about the improved sanitation behavior among respondents in the intervention and counterfactual groups. Trained qualitative researchers will conduct in-depth interviews and focus group discussions with purposively selected respondents in intervention clusters to assess their interaction and experience with the Nam Nalavazhvu intervention. We will use semistructured questionnaires, memos, and note-taking to record observations and will record interviews as required. Verbal consent will be taken before every data collection activity except for observations made in public spaces. Results from this qualitative investigation will be used to interpret the impact of the intervention.

### Data Management

All survey data will be transmitted through secured servers and stored in password-protected folders in the Penn+Box (The University of Pennsylvania). To protect confidentiality, all subjects will be deidentified for analysis. Data will only be accessible to University of Pennsylvania faculty, staff, and data management personnel.

### Statistical Analyses

We will use intention-to-treat analyses to assess the difference in specific outcomes between study arms after exposure to 1 year of the Nam Nalavazhvu intervention. For most of the outcomes, we will use a log-binomial regression model to assess prevalence ratios of postintervention sanitation-related outcomes across intervention groups. We will consider adjusting for variables that were imbalanced between the groups at baseline in adjusted models. We will also use generalized estimating equations with robust SEs to account for the clustering of observations within each cluster (ward). We will use postestimation commands to estimate and report the average marginal effects. We will not adjust *P* values based on multiple comparisons.

In additional analyses, we will use appropriate multivariate models to assess the impact of the intervention on secondary outcomes. Both unadjusted and adjusted effect estimates will be reported for all outcomes. Following the process evaluation findings, if fidelity or intervention quality are found to vary considerably in the trial, we will consider a per-protocol or other appropriate analysis to assess the impact of the Nam Nalavazhvu intervention on our outcomes of interest. The analyses will be conducted by the scientific team (SA, AS, UD, JK, CB) with statistical software including R (The R Project for Statistical Computing) and Stata (StataCorp).

Qualitative data will be collected until saturation is reached. We will validate key findings using triangulation of data across multiple data sources. We plan to conduct gender-stratified analyses to understand challenges to adoption of exclusive toilet usage.

### Ethics and Dissemination

The ethics review board at the University of Pennsylvania (institutional review board protocol no. 833854) and the Catalyst Foundation in India reviewed and approved this research protocol. The trial is registered with ClinicalTrials.gov (NCT04269824). All amendments and protocol modifications will be updated there. All enrolled study communities provided verbal consent to enroll in the study. Surveyed individuals provided informed consent. This consent process was conducted in the local language, Tamil. Participants will receive messages that may encourage them to improve their sanitation conditions or practices. Our assessment is that the benefits to study participation outweigh the minimal risks. Deidentified data will be used during analysis.

Research findings will be disseminated through presentations at conferences and submitted to peer-reviewed journals for open access. Our results will be shared with relevant local stakeholders through community-based meetings in participating wards through presentations made to the district and state level officials in Tamil Nadu.

### Data Monitoring, Reporting Harms, and Auditing

The research team (SA, KC, AS, UD) has text message groups and weekly calls with the implementation partner to discuss progress and issues from the field, including adverse events, so that prompt action can be taken. No harm is anticipated to the intervention recipients in this study. There are no plans for a data monitoring committee or audits for this trial.

## Results

This study completed its baseline survey in January 2020. Endline assessments are planned for July 2021. Results are anticipated to be published by the end of 2022.

## Discussion

This study will evaluate the effectiveness of the Nam Nalavazhvu intervention, a demand-side, descriptive norm- and network-centric intervention approach that aims to shift empirical expectations on targeted sanitation behaviors. Our behavior change communication approach employs dynamic signaling (ie, dissemination of descriptive information regarding others’ actual or intended improved sanitation practices) and reflects a strategy that is novel to the sanitation sector but which has been effective in changing a variety of behaviors, such as water use, drinking behavior, and energy consumption [36,44-46]. Evaluating the impact of such an approach may have widespread implications for policy and practice for sanitation programs in India and beyond if the intervention proves effective in improving sanitation behaviors via changing people’s empirical expectations. Our plan to also track normative expectations will also allow us to determine whether an intervention focused on shifting of empirical expectations has spillover effects on normative expectations.

Evidence from this study will address knowledge gaps regarding the application and effectiveness of a norm-diagnostic approach in the design of behavior change strategies that intervene upon the social determinants of collective sanitation behaviors. The intervention uses outreach workers and social media users to deploy most of its messages. Understanding the transmission of messages during household visits, peer learning sessions, and text messages will inform recommendations on the feasibility and effectiveness of using these platforms for norm-centric interventions. Insights generated in this study may generally contribute to the tools available to address descriptive norms for community-based interventions.

Limitations of this CRT include the use of wards as clusters in peri-urban communities. Although these are the smallest geographic operational units, some boundaries in specific districts were redrawn following the start of the intervention, leading to concerns about spillovers across buffer areas. Two critical country-specific incidences are impacting the implementation of the interventions. One is the highly contentious Citizens Amendment Act passed in December 2019 that led to nationwide protests in India. In our study, it led to refusals by households in predominantly Muslim wards, who resisted participating in any study that includes survey-based instruments. Secondly, the ongoing COVID-19 pandemic has led to considerable interruption in the implementation of group-level intervention activities. These aspects are being assessed through our process evaluation and will inform the interpretation of the results from this CRT.

## Appendix

Multimedia Appendix 1 Study wards enrolled in each town panchayat, Tamil Nadu, India, 2020.

Multimedia Appendix 2 Outcome measures.

## Abbreviations

BCD: Behavior Centered Design
CLTS: Community-Led Total Sanitation
CRT: cluster-randomized controlled trial
CSNBD: Center for Social Norms and Behavioral Dynamics
LENNS: Longitudinal Evaluation of Norms and Network Study
RANAS: Risks, Attitudes, Norms, Abilities, and Self-regulation
SBM: Swachh Bharat Mission
SNT: social norms theory
WHO-5: The 5-item World Health Organization Well-Being Index
