# Supplementary material for: Norms and Social Network–Centric Behavior Change Intervention (Nam Nalavazhvu) for Improved Toilet Usage in Peri-Urban Communities of Tamil Nadu: Protocol for a Cluster-Randomized Controlled Trial
Source: JMIR Res Protoc. 2021 May 3;10(5):e24407. doi: 10.2196/24407 (PMC8129879; doi:10.2196/24407)
Supplement: Multimedia Appendix 1 [file resprot_v10i5e24407_app1.docx]

**Table S1: Number of wards selected from each town panchayat, Tamil Nadu, India, 2020**

| **District** | **Town panchayat** | **Number of treatment wards** | **Number of counterfactual wards** |
| --- | --- | --- | --- |
| Pudukkottai | Ponnamaravathi | 4 | 4 |
|  | Arimanagalam | 4 | 4 |
|  | Keeranur | 4 | 4 |
|  | Alangudi | 4 | 3 |
|  | Keeramangalam | 3 | 4 |
| Karur | Nangavaram | 4 | 4 |
|  | Puliyur | 4 | 4 |
|  | Uppidamangalam | 4 | 4 |
|  | Punjai Thottakurichi | 3 | 3 |
|  | PJ Cholapuram | 4 | 4 |
|  | Total | 38 | 38 |

| Table S2: Key indicators used to assess sanitation practices, beliefs, expectations, and behavioral outcomes | | | |
| --- | --- | --- | --- |
| **Outcome** | **Indicator** | **Questions** | **Type of data** |
| Sanitation Outcomes | Access to Toilets | *Do your household members have access to any toilet facility?* | Reported and spot observation |
|  |  | *How many toilets do your household members have access to?*  *Spot observation to categorize type of toilets (JMP definitions*^1^*)* |  |
|  | Toilet Ownership | *Who owns the toilet you use primarily?* | Reported |
|  |  | *Out of 10 households with toilets in your community, how many do you think own a toilet?* |  |
|  | Toilet Use | *Where did you defecate the last time you needed to?* | Respondent reported for self and family members  Spot observation for signs of use |
|  |  | *During the last two days, where was your primary place of defecation?* |  |
|  |  | *During the last two days, how often did you defecate in the open?* |  |
|  |  | *During the last two days, when you needed to defecate, how often did you use a toilet?* |  |
|  |  | *During the last 7 days including today, did you use a toilet for defecation every time?* |  |
|  |  | *Did your primary place of defecation change over the course of the year?* |  |
|  | Toilet cleaning/maintenance | *During the last 7 days, including today, how many times was this toilet cleaned?*  *In the past one year, have you made any repairs to the toilet? (i.e., have you fixed anything that became broken, damaged, or worn out on this toilet)*  *In the past one year, have you added or improved anything on this toilet to upgrade it?* | Reported  Supplemented by spot observation of toilet facilities |
|  | Empirical Expectations | *Out of 10 households with toilets in your community, how many do you think own a toilet?* | Reported^2^ |
| Beliefs and Expectations |  | *Think about ten members of your community. Out of them, how many do you think use a toilet every time to defecate?* |  |
|  |  | *Think about members of your community who don’t have a private toilet. Out of them, how many do you think used a community latrine the last time they needed to defecate?* |  |
|  |  | *Out of 10 households with toilets in your community, how many do you think keep it clean?* |  |
|  | Temporal changes | *Think about your community 3 months ago. Do more, fewer or about the same number of people in your community use a toilet every time now when they need to defecate?* | Reported |
|  |  | *In the next 3 months, do you think there will be fewer, more, or about the same number of people who will use toilet every time when they need to defecate?* |  |
|  | Personal Normative Beliefs | *Some people who have a toilet still defecate in the open. Society may think that this is right or wrong. Do you personally think it’s right, wrong, or neither for someone who has a toilet, to defecate in the open?* | Reported |
|  |  | *Some people who defecate in the open do not have a toilet. Society may think that this is right or wrong. Do you personally think it’s right, wrong, or neither for someone who does not have a toilet, to defecate in the open?* |  |
|  | Normative Expectations | *Out of ten members of your community, how many do you think believe one should use a toilet to defecate?* | Reported |
|  |  | *Do you disagree, somewhat disagree, neither agree nor disagree, somewhat agree or agree with the following statement: “Members of my community think it is acceptable to defecate in the open”* |  |
|  | Externalities and Sanctions | *Think about other people defecating in the open. Can this negatively affect you, personally?* | Reported |
|  |  | *If someone from your community defecated in the open, would anyone do or say anything in response to that?* |  |
|  | Causal vignette | *Please imagine an area similar to where you live. Someone from your area, whom you don’t know, moved there one year ago. He/she has access to both a toilet, and a field he/she could use to defecate. He/she learned that [most]/[few] people disapprove of defecating in the open [and]/[, but] he/she also learned that [most/few] people do it. What do you think he/she will do?* | Reported |
|  | Health outcomes |  |  |
|  |  | *Mental wellbeing*  *Diarrhea prevalence*  *Respiratory illness* | World Health Organization- Five Well-Being Index (WHO-5)^3^  3- day recall (at least 3 or more loose or liquid stools per day)^4^  3- day recall of presence of cough and/or shortness of breath/difficulty* |
|  |  |  |  |

**Primary caregivers will be asked to report for children*

*The complete questionnaire and all other surveys used in this trial is available on request.*

1. WHO/UNICEF. *WHO | Progress on Drinking Water, Sanitation and Hygiene*. World Health Organization; 2017. http://www.who.int/water_sanitation_health/publications/jmp-2017/en/. Accessed August 22, 2017.

2. Bicchieri C. *Norms in the Wild*. Oxford University Press; 2017. doi:10.1093/acprof:oso/9780190622046.001.0001

3. World Health Organisation. Wellbeing Measures in Primary Health Care/ The Depcare Project. *Rep a WHO Meet*. 1998.

4. WHO IMCI. Integrated Management of Childhood Illness (IMCI) Chart Booklet. *Distance Learn Course*. 2014.

S3: Informed consent form

*Individuals enrolled specifically for the assessment will be invited to take a survey. The enumerator will read out the following consent form to confirm their consent.*

***Title of the Research Study: Longitudinal evaluation of norms and networks study (LENNS)***

Hello (Namaskara). My name is ___________ and I work for Kantar Public in Delhi on behalf of the University of Pennsylvania in the USA. We are conducting research on improving people’s wellbeing by encouraging them to improve their sanitation related practices. We want to learn more about what impacts people’s behaviors when we inform them about others in their communities. Your ward may or may not receive the intervention based on a lottery, but we would like to invite you to participate in a survey as a part of the research study. To be clear, I am not from the government.

You are being asked to join this study because you are a resident of Tamil Nadu and you are above 18 years of age. This interview will be at or near your home in a quiet and private place to give you more privacy and to hear you better. The survey will last approximately 60 to 90 minutes. If you agree to participate, we will also conduct a follow-up survey after one year.

Participation in this survey is voluntary.  You can ask me any questions, choose to not answer specific questions and end this interview at any time. There is no benefit or loss to you if you decide to drop out of the survey. Your participation in the overall study is not affected by your participation in this survey. You can still receive study related messages and visits even if you decline participating in this survey.

The risks of participating in the survey are minimal. You may feel uncomfortable by the personal nature of some questions.  Your participation in the survey will have no effect on your eligibility to participate in other programs. You will not be paid or get direct or indirect benefits for participating in this study. Our findings may eventually help improve health in communities like yours.

We will take all reasonable steps to ensure your privacy. No one will know that we interviewed you specifically. You or any other participants cannot be personally identified through this research and we will not use your name or address in the results of the study. The information of this survey will be combined with other data and made publicly available for other researchers to study.

During this study, we are required by law to report any information that comes to use about child abuse, intent to harm self or others to the concerned authorities.

***Contact information:***
You will receive a copy of this consent letter. You can ask me any questions but if you have additional questions regarding your participation in this study, you may speak with:

Dr. Upasak Das, Researcher, Email: upasak.das@gmail.com; Phone: +91 956455420

Peter McNally, Project Manager, Email: [mcnally@sas.upenn.edu](mailto:mcnally@sas.upenn.edu); If you want to talk to someone not working on the study, you may contact Sourav Jena (IRB Coordinator) from the SRI -Institutional Review Board at 011 – 26851660.

Thank you for your cooperation I am willing to provide consent for a follow up which is going to take part approximately 12 months from now

YES______ NO______

____________________ ____________________

Signature of Participant Signature of Interviewer
