# Supplementary material for: Norms and Social Network–Centric Behavior Change Intervention (Nam Nalavazhvu) for Improved Toilet Usage in Peri-Urban Communities of Tamil Nadu: Protocol for a Cluster-Randomized Controlled Trial
Source: JMIR Res Protoc. 2021 May 3;10(5):e24407. doi: 10.2196/24407 (PMC8129879; doi:10.2196/24407)
Supplement: Multimedia Appendix 2 [file resprot_v10i5e24407_app2.docx]

| Table S2: Key indicators used to assess sanitation practices, beliefs, expectations, and behavioral outcomes | | | |
| --- | --- | --- | --- |
| **Outcome** | **Indicator** | **Questions** | **Type of data** |
| Sanitation Outcomes | Access to Toilets | *Do your household members have access to any toilet facility?* | Reported and spot observation |
|  |  | *How many toilets do your household members have access to?*  *Spot observation to categorize type of toilets (JMP definitions*^1^*)* |  |
|  | Toilet Ownership | *Who owns the toilet you use primarily?* | Reported |
|  |  | *Out of 10 households with toilets in your community, how many do you think own a toilet?* |  |
|  | Toilet Use | *Where did you defecate the last time you needed to?* | Self-reported  Proxy reported for family members if unavailable  Spot observation for signs of use |
|  |  | *During the last two days, where was your primary place of defecation?* |  |
|  |  | *During the last two days, how often did you defecate in the open?* |  |
|  |  | *During the last two days, when you needed to defecate, how often did you use a toilet?* |  |
|  |  | *During the last 7 days including today, did you use a toilet for defecation every time?* |  |
|  |  | *Did your primary place of defecation change over the course of the year?* |  |
|  | Toilet cleaning/maintenance | *During the last 7 days, including today, how many times was this toilet cleaned?*  *In the past one year, have you made any repairs to the toilet? (i.e., have you fixed anything that became broken, damaged, or worn out on this toilet)*  *In the past one year, have you added or improved anything on this toilet to upgrade it?* | Reported  Supplemented by spot observation of toilet facilities |
|  | Empirical Expectations | *Out of 10 households in your community, how many do you think own a toilet?* | Reported^2^ |
| Beliefs and Expectations |  | *Think about ten members of your community. Out of them, how many do you think use a toilet every time to defecate?* |  |
|  |  | *Think about members of your community who don’t have a private toilet. Out of them, how many do you think used a community latrine the last time they needed to defecate?* |  |
|  |  | *Out of 10 households with toilets in your community, how many do you think keep it clean?* |  |
|  | Temporal changes | *What do you think about the following statement [5 point- Likert scale]?*  *“I think more people use a toilet in my ward compared to six months ago”* | Reported |
|  |  | *“I think more people expect others to use toilets compared to six months ago”* |  |
|  | Personal Normative Beliefs | *Some people who have a toilet still defecate in the open. Society may think that this is right or wrong. Do you personally think it’s right, wrong, or neither for someone who has a toilet, to defecate in the open?* | Reported |
|  |  | *Some people who defecate in the open do not have a toilet. Society may think that this is right or wrong. Do you personally think it’s right, wrong, or neither for someone who does not have a toilet, to defecate in the open?* |  |
|  | Normative Expectations | *Out of ten members of your community, how many do you think believe one should use a toilet to defecate?* | Reported |
|  |  | *What do you think about the following statement [5 point- Likert scale]: “Members of my community think it is acceptable to defecate in the open”*  *“I think my neighbors should use a toilet”* |  |
|  | Externalities and Sanctions | *Think about other people defecating in the open. Can this negatively affect you, personally?* | Reported |
|  |  | *If someone from your community defecated in the open, would anyone do or say anything in response to that?* |  |
|  | Causal vignette | *Please imagine an area similar to where you live. Someone from your area, whom you don’t know, moved there one year ago. He/she has access to both a toilet, and a field he/she could use to defecate. He/she learned that [most]/[few] people disapprove of defecating in the open [and]/[, but] he/she also learned that [most/few] people do it. What do you think he/she will do? [Response: Likely use a toilet, Either way, Unlikely to use a toilet]* | Reported |
|  | Health outcomes |  |  |
|  |  | *Mental wellbeing*  *Diarrhea prevalence*  *Respiratory illness* | World Health Organization- Five Well-Being Index (WHO-5)^3^  7- day recall (at least 3 or more loose or liquid stools per day)^4^  7- day recall of presence of cough and/or shortness of breath/difficulty breathing* |
|  |  |  |  |

**Primary caregivers will be asked to report for children*

*The complete questionnaire and all other surveys used in this trial is available on request.*

1. WHO/UNICEF. *WHO | Progress on Drinking Water, Sanitation and Hygiene*. World Health Organization; 2017. http://www.who.int/water_sanitation_health/publications/jmp-2017/en/. Accessed August 22, 2017.

2. Bicchieri C. *Norms in the Wild*. Oxford University Press; 2017. doi:10.1093/acprof:oso/9780190622046.001.0001

3. World Health Organisation. Wellbeing Measures in Primary Health Care/ The Depcare Project. *Rep a WHO Meet*. 1998.

4. WHO IMCI. Integrated Management of Childhood Illness (IMCI) Chart Booklet. *Distance Learn Course*. 2014.
